# Understanding the evolution of viviparity using intraspecific variation in reproductive mode and transitional forms of pregnancy

**DOI:** 10.1111/brv.12836

**Published:** 2022-01-30

**Authors:** Camilla M. Whittington, James U. Van Dyke, Stephanie Q. T. Liang, Scott V. Edwards, Richard Shine, Michael B. Thompson, Catherine E. Grueber

**Affiliations:** 1 School of Life and Environmental Sciences, The University of Sydney, Heydon-Laurence Building A08, Sydney, NSW, 2006; 2 Department of Pharmacy and Biomedical Sciences, School of Molecular Sciences, La Trobe University, Building 4, Wodonga, VIC, 3689, Australia; 3 Department of Organismic and Evolutionary Biology, Harvard University, and Museum of Comparative Zoology, Cambridge, MA, 02138, U.S.A.; 4 Department of Biological Sciences, Macquarie University, North Ryde, NSW, 2109, Australia

**Keywords:** bimodal reproduction, evolutionary innovations, oviparity, reproductive biology, squamate

## Abstract

How innovations such as vision, flight and pregnancy evolve is a central question in evolutionary biology. Examination of transitional (intermediate) forms of these traits can help address this question, but these intermediate phenotypes are very rare in extant species. Here we explore the biology and evolution of transitional forms of pregnancy that are midway between the ancestral state of oviparity (egg-laying) and the derived state, viviparity (live birth). Transitional forms of pregnancy occur in only three vertebrates, all of which are lizard species that also display intraspecific variation in reproductive phenotype. In these lizards (*Lerista bougainvillii, Saiphos equalis, and Zootoca vivipara*), geographic variation of three reproductive forms occurs within a single species: oviparity, viviparity, and a transitional form of pregnancy. This phenomenon offers the valuable prospect of watching ‘evolution in action’. In these species, it is possible to conduct comparative research using different reproductive forms that are not confounded by speciation, and are of relatively recent origin. We identify major proximate and ultimate questions that can be addressed in these species, and the genetic and genomic tools that can help us understand how transitional forms of pregnancy are produced, despite predicted fitness costs. We argue that these taxa represent an excellent prospect for understanding the major evolutionary shift between egg-laying and live birth, which is a fundamental innovation in the history of animals.

## I. INTRODUCTION

A major focus of evolutionary biology is understanding the origin of new structures (e.g. eyes, wings, flowers) and abilities (e.g. vision, flight, sexual reproduction) (e.g. Shubin, Tabin & Carroll, 2009; Wagner, 2015). It is important to understand these processes because such innovations underpin much of the diversity of life on Earth. We now know from evolutionary developmental biology and palaeontology that novelties emerge in stages (Pieretti *et al*., 2015). The tricky part is understanding *how* new structures and functional innovations evolve, because the process can take many generations. To watch evolution in action, we should ideally examine transitional forms to discover how genetic changes have enabled the emergence of these traits. Here, we explore how transitional forms of pregnancy can help us understand the evolution of the major transition from egg-laying to live birth in vertebrates.

One of the most fundamental aspects of animal reproduction is parity mode: whether females lay eggs (oviparity) or give birth to live young (viviparity) (Blackburn, 2015a). In oviparity, eggs are fertilised externally or embryos are laid within eggs, early in development, and complete much of their development external to the parent (Blackburn, 2000). By contrast, viviparous females incubate embryos internally inside the reproductive tract until development is complete (Blackburn, 2000). Viviparity is a compelling example of convergent evolution because it has evolved independently from the ancestral state of egg-laying more than 150 times in vertebrates as diverse as fish, amphibians, reptiles, and mammals (Blackburn, 2015a), and many more times in invertebrates (Ostrovsky *et al*., 2016). The factors underpinning reproductive mode are complex, and evolutionary transitions from oviparity to viviparity involve many changes to anatomy, physiology, behaviour and genetics in support of internally incubated embryos (Blackburn, 2006, 2015a; Murphy & Thompson, 2011).

The repeated emergence of viviparity in diverse taxa offers naturally replicated evolutionary ‘experiments’ to test hypotheses about the biology and evolution of this important trait. This review highlights the utility of a handful of unique species that offer exceptional opportunities for us to understand how viviparity has evolved. We focus on species (all squamate reptiles) with two unusual reproductive features: intraspecific variation in reproductive mode, and transitional forms between oviparity and viviparity. This situation allows us to observe ‘evolution in action’ to help determine the mechanistic basis and understand the drivers of the transition between egg-laying and live birth.

## II. THE EVOLUTION OF VERTEBRATE VIVIPARITY

Although viviparity has evolved independently hundreds of times in animals, some taxa offer better opportunities to understand this phenomenon than others. While nearly all mammals are viviparous, pregnancy in this group is an ancient trait that evolved only once: viviparous therians last shared a common ancestor with egg-laying metatherians 191–163 million years ago (Mya) (dos Reis *et al*., 2012). It is therefore difficult to reconstruct the evolutionary sequence leading to viviparity in mammals because changes that produced intermediate steps are likely to have been lost over time (Blackburn, 2006; Van Dyke, Brandley & Thompson, 2014). By contrast, viviparity has evolved at least 22 times in extant fishes, eight times in amphibians, and 115 times in squamate reptiles (lizards, snakes, amphisbaenians), at various times in evolutionary history, providing rich opportunities for comparative research (Stewart & Blackburn, 2014; Blackburn, 2015a).

Squamates exhibit more origins of viviparity than any other vertebrate taxon (Blackburn, 2006, 2015a; Stewart & Blackburn, 2014), including some comparatively recent transitions, meaning that there are many closely related oviparous and viviparous species that are ideal for evolutionary studies of reproduction (Van Dyke *et al*., 2014). Squamates also are unique amongst vertebrates in displaying intraspecific variation in reproductive mode, referred to here as ‘bimodal reproduction’. Such species typically display both oviparity and viviparity in different geographic locations. Bimodally reproductive species are extremely rare, but probably represent the most recent origins of vertebrate viviparity. Estimates of origins of viviparity in bimodally reproductive species range from ~4.5 Mya in the European lizard *Zootoca vivipara* (Lichtenstein 1823) (Cornetti *et al*., 2014) to just 0.01 Mya in the Australian lizard *Lerista bougainvillii* (Gray 1839) (Qualls & Shine, 1996). Furthermore, some squamates display transitional forms between the two reproductive modes (see Section III.2), providing further opportunities to understand the steps in the evolution of viviparity.

## III. VARIATION IN SQUAMATE REPRODUCTIVE MODE

The major functional difference between oviparous and viviparous species is the timing of expulsion of embryos from the female reproductive tract. ‘Parition’ describes both parturition (the process of giving birth to live young) and oviposition (the process of laying eggs) (Blackburn, 1992). In oviparity, parition occurs before the embryos have completed development and eggs are incubated externally. In viviparous squamates, parition occurs only after the embryos have completed development. Squamate viviparity may have evolved *via* gradual increases in the duration of egg retention inside the body of the mother (Packard, Tracy & Roth, 1977; Shine & Bull, 1979; Andrews & Rose, 1994; Andrews & Mathies, 2000; Blackburn, 2006). Non-gradualistic models (particularly, evolution *via* punctuated equilibrium) have also been proposed to account for the relative lack of intermediate forms (see Section III.2) (Blackburn, 1995, 2006, 2018; Stewart, 2013; Stewart & Blackburn, 2014). One peculiarity of squamate viviparity is that most viviparous species are lecithotrophic, meaning that females ovulate large, yolk-filled eggs similar to those of their oviparous relatives. This situation is in contrast to the matrotrophy exhibited by viviparous mammals, in which mothers transport substantial quantities of nutrients to developing embryos (Van Dyke & Griffith, 2018). With few exceptions, viviparous squamates have only simple placentae that provide embryonic gas exchange, water transport, and limited nutrient transfer (Thompson, Stewart & Speake, 2000; Stewart & Blackburn, 2014).

Egg-laying and live-bearing squamates differ in several important ways. For example, oviparous mothers possess specialised structures such as uterine glands that lay down calcareous eggshell (from which developing embryos derive some calcium) (Heulin *et al*., 2005), and must find suitable laying sites for eggs to develop in the external environment (Shine, 2015). By contrast, the egg coverings of viviparous species are very thin (or absent), lack a calcareous layer, and are broken by neonates at or soon after birth. Because the eggshell impedes efficient transport of gases and other molecules between the mother and embryo, thinning or loss of egg coverings has likely been selected for in viviparous parents (Blackburn, 2005, 2006; Shine & Thompson, 2006). Thus, viviparous mothers lack or have reduced anatomical structures involved in eggshell production (e.g. Heulin *et al*., 2005; Braz *et al*., 2018), and provide calcium to embryos *via* a placenta and in the yolk (Linville *et al*., 2010). Species in five skink genera also transport substantial quantities of other nutrients to developing embryos via the placenta (termed obligate placentotrophy), although the condition is otherwise rare (reviewed in Thompson & Speake, 2006; Van Dyke & Griffith, 2018). Comparisons of viviparous and oviparous squamates have revealed differences in metabolic costs of reproduction (e.g. Foucart *et al*., 2014), endocrine activity (e.g. Paul *et al*., 2020), histology of gestational tissues (e.g. Guillette Jr. & Jones, 1985; Adams *et al*., 2007; Braz *et al*., 2018), and behaviour and performance (e.g. Recknagel & Elmer, 2019), which may be underpinned by differences in regulation of gene expression between reproductive modes (e.g. Griffith *et al*., 2016, 2017; Gao *et al*., 2019; Foster *et al*., 2020).

The fact that viviparity has evolved so many times in squamates suggests that the taxon may possess exaptations enabling frequent transitions to viviparity. For example, traits such as internal fertilisation, extended egg retention, vascularised oviducts, and substantial calcium supply in the yolk (rather than some reliance on eggshell-derived calcium) may have predisposed squamates to the evolution of viviparity (Packard *et al*., 1977; Blackburn, 2006; Blackburn & Stewart, 2011). There is likely an evolutionary advantage to squamates being able to give birtht olive young in some conditions. For example, viviparity may have enabled species to exploit new habitats, such as cold climates (e.g. Weekes, 1935; Tinkle & Gibbons, 1977; Shine, 2004, 2014; Esquerré *et al*., 2019; Recknagel, Kamenos & Elmer, 2021b). Viviparity also allows mothers to protect developing embryos from predators and regulate optimal conditions for their growth (e.g. Shine, 1995; Webb, Shine & Christian, 2006; Blackburn & Stewart, 2011). Compared to oviparous mothers that do not engage in parental care, viviparous mothers may have a greater ability to manipulate the gestational environment to maximise the fitness of offspring. For example, mothers of some viviparous species can influence offspring sex (Robert & Thompson, 2001; Wapstra *et al*., 2004; Allsop et al., 2006; Ji *et al*., 2006; Zhang *et al*., 2010; Tang *et al*., 2012), which could produce the rarer sex in a sex-biased population, and thereby increase offspring reproductive success or influence offspring morphology or viability (Shine & Harlow, 1993). Viviparous mothers can also reproduce in situations where there are no suitable sites for egg-laying e.g. aquatic environments (Blackburn & Stewart, 2011; Stewart & Blackburn, 2014). However, viviparity also incurs costs: while Qualls & Shine (1998a) found no significant difference in cost between oviparous and viviparous reproduction, others measured increased energetic costs of pregnancy (Robert & Thompson, 2000; Foucart et al., 2014), increased susceptibility to predation, reduced opportunities for reproduction within a given active season (Tinkle & Gibbons, 1977), and mother–offspring conflict over resource allocation(e.g. Crespi & Semeniuk, 2004) in viviparous species. Viviq arous mothers may also have lower fecundity (Recknagel & Elmer, 2019), and carry embryos for longer, incurring locomotory costs and potentially making them vulnerable to predation (Shine, 1980; Qualls & Shine, 1995). Reduced water allocation to embryos in viviparous squamates mean that total mass and volume of an egg and a full-term embryo are similar, hence reducing or eliminating the additional burden that might have been expected from uterine retention of larger embryos in viviparous taxa (Meiri *et al*., 2020). To determine the evolutionary conditions that are likely to give rise to viviparity, the costs and benefits of each reproductive mode are ideally measured in taxa that are very closely related, but which have different parity modes (Qualls & Shine, 1995; Andrews & Mathies, 2000; Stewart, 2013; Van Dyke et al., 2021). Bimodally reproductive species meet these conditions perfectly.

### (1) Bimodal reproduction

Squamates are the only vertebrates that exhibit so much variation in the timing of parition that both viviparity and oviparity occur in a single species (bimodal reproduction). Nevertheless, bimodal reproduction is rare: of the approximately 11,000 extant squamate species (Uetz, Freed & Hošek, 2020), only ten have been reliably described as bimodally reproductive (reviewed in Blackburn, 2015b). These are the lizards *Glaphyromorphus nigricaudis* (Macleay, 1877), *Lerista bougainvillii* (Gray 1839), *Madascincus igneocaudatus* (Brygoo, 1981), *Saiphos equalis* (Gray 1825), *Trachylepis capensis* (Gray 1831), and *Zootoca vivipara*, and the snakes *Echis carinatus* (Schneider, 1801), *Helicops angulatus* (L. 1758), *Protobothrops jerdonii* (Günther, 1875), and *Psammophylax variabilis* (Günther, 1893) [see Blackburn, 1985 and Shine, 1985 for analyses of reproductive bimodality in each species; Blackburn, 1999]. An additional two species [*Trachylepis damarana* (Peters 1870), *Trachylepis occidentalis* (Peters 1867)] may be bimodal, but monophyly of oviparous and viviparous populations still needs to be confirmed (Weinell *et al*., 2019). Despite Blackburn (2015b) considering these species to be ‘reliably’ described as bimodal, the taxonomy of some of them may not be robust due to a relative lack of research. Furthermore, some, like *E. carinatus* and *P. jerdonii*, have large distributions over varied environments, including rainforest, desert, and alpine areas (McDiarmid, Campbell & Toure, 1999). Thus it is possible that at least some of these bimodal ‘species’ are in fact species complexes, with cryptic allopatric speciation across geographic boundaries that have not yet been identified. Further research is required to confirm reproductive bimodality for most of these species, except for those for which we have robust evidence.

The evidence for reproductive bimodality is strongest for *Z. vivipara* (e.g. Lantz, 1927; Panigel, 1956), *L. bougainvillii* (Greer, 1989; Qualls *et al*., 1995), *S. equalis* (Smith & Shine, 1997; Smith, Austin & Shine, 2001), and H. angulatus (e.g. Mole, 1924; Rossman, 1973), for which there are several to many peer-reviewed publications (see Section IV) documenting the evidence of their bimodality (Whittington, 2021), whereas the evidence for the other taxa listed above is limited to a few difficult-to-verify reports that rely on less-detailed phylogenetic information. Where it is confirmed, reproductive bimodality within a species presents a unique opportunity to study the evolution of reproductive strategies, because oviparous and viviparous individuals can be compared without the confounding effects of speciation (Qualls & Shine, 1995, 1998b; Smith & Shine, 1997; Smith *et al*., 2001; Heulin *et al*., 2002; Shine, 2014; Whittington, 2021). In these species, the distribution of individuals with different reproductive (parity) modes varies geographically over their range (Smith & Shine, 1997; Qualls & Shine, 1998b; Horreo *et al*., 2019), such that variation in parity mode does not always coincide with speciation (Blackburn, 2015a).

### (2) Transitional forms between oviparity and viviparity

Most oviparous squamates lay eggs at a much later stage of embryonic development than do other oviparous reptiles. For example, tuatara and turtles (both non-squamates) oviposit when embryos are at the gastrula stage, and crocodiles (non-squamates) lay eggs at the neurula stage (Andrews & Mathies, 2000). By contrast, most oviparous squamates lay eggs about one third of the way through development, at around the time the limb buds develop (Shine, 1983; Blackburn, 1995; Andrews & Mathies, 2000). Note that while some derived chameleons oviposit at the gastrula stage (Andrews & Mathies, 2000; Andrews, 2004), the ancestral chameleon likely oviposited at the limb-bud stage; viviparous chameleons are nested within ‘typical’ oviparous clades (Andrews & Karsten, 2010; Hughes & Blackburn, 2020). The late deposition of most oviparous squamate embryos is a potential exaptation for viviparity in this lineage (Andrews & Mathies, 2000). Still, there are a number of examples of live birth in extinct reptile lineages, suggesting that pregnancy may have ancient origins within the non-squamate reptiles as well (e.g. Cheng, Wu & Ji, 2004; Blackburn & Sidor, 2014; Liu *et al*., 2017).

**Fig. 1.**
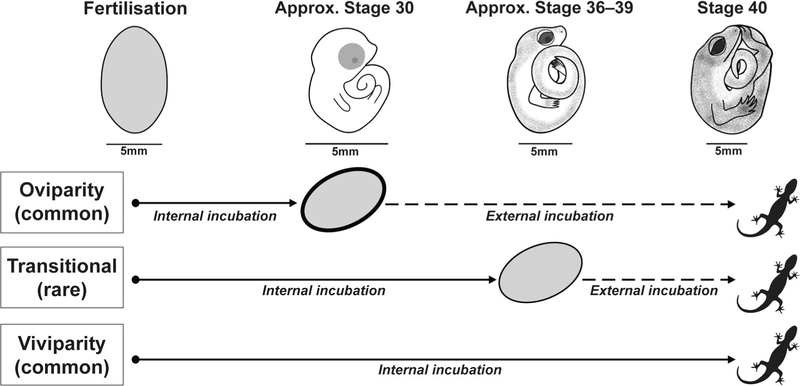
Embryonic development in squamates with different reproductive modes. The ‘transitional’ phenotype is indicative of some populations of *Lerista bougainvillii* and *Saiphos equalis* (to a lesser extent, also *Zootoca vivipara*) which produce very thinly shelled eggs at a late developmental stage, intermediate between oviparity and viviparity (see Section III.2 for details). Schematics of embryonic stages are based on the staging table of *Zootoca vivipara* by Dufaure & Hubert (1961); the yolk is not shown, except for the image of the fertilised egg. Approximate embryonic sizes are indicated (Dufaure & Hubert, 1961).

In lizards, embryonic development is divided into 40 stages (Dufaure & Hubert, 1961). Stage 40 is complete development, which is when viviparous squamates are born and oviparous squamate eggs hatch (Fig. 1). Variation in the degree of offspring development at the time of hatching or birth may be biologically significant in squamates, but does not involve marked variation in offspring morphology (e.g. Olsson *et al*., 1996; Shine & Olsson, 2003). The embryonic development mode at oviposition in squamates is stage 30. This is about one third of the way through development because the staging system is not linear with time; 80% of developmental stages are achieved in the first half of gestation (Shine, 1983; Andrews & Mathies, 2000). Despite the comparatively ‘late’ developmental stage of squamate eggs at laying, very few oviparous squamates lay eggs after stage 35 (the end of the limb-bud stages) (Shine, 1983; Blackburn, 1995; Andrews & Mathies, 2000). This is the point when embryonic mass increases rapidly relative to hatchling mass, and metabolic rate, water, and oxygen demands are highest (reviewed in Andrews & Mathies, 2000). ‘Late’ oviposition compared to most other oviparous squamates is therefore an intermediate phenotype, or transitional form, between oviparity and viviparity.

Some squamates exhibit condition-dependent variation in egg retention. For example, in some oviparous *Sceloporus* spp., eggs can be retained to later stages of development in response to arid conditions that are unsuitable for oviposition (e.g. Mathies & Andrews, 1996; Andrews & Mathies, 2000; García-Collazo *et al*., 2012). *Opheodrys vernalis* (Harlan, 1827) eggs also have condition-dependent variation in incubation duration (Blanchard, 1933). Eggs of *Lacerta agilis* (L. 1758) from the northern (cold-climate) extreme of the species’ range have widely varying inter-clutch incubation periods, with eggs that are laid later in the season hatching after briefer periods (Shine, Wapstra & Olsson, 2018). An experimental island population in this region has even more variable incubation periods, although embryonic stage at oviposition is not yet known (Olsson *et al*., 2018). Oviparous populations of the bimodally reproductive H. angulatus may also exhibit a range of different incubation durations [from 17 (Rossman, 1973) to 40 (Ford & Ford, 2002) to 109 (Gorzula & Señaris, 1998) days]. The shorter incubation durations could indicate a transitional form of pregnancy (Braz, Scartozzoni & Almeida-Santos, 2016), or alternatively could be a result of unsuitable nesting conditions (Ford & Ford, 2002). The latter explanation seems likely, given the small sample sizes for these studies (1 or 2 clutches each). Therefore, additional data (incubation duration, embryonic stage at oviposition) from multiple clutches in different regions are required to determine whether *H. angulatus* truly exhibits a transitional form of pregnancy.

Detailed studies on a montane oviparous skink in south-eastern Australia, the three-lined skink (*Bassiana duperreyi*, also called *Acritoscincus duperreyi*, Gray 1838), illustrate how the duration of retention of eggs *in utero* (and thus, embryonic stage at hatching) can be driven simultaneously by local adaptation and by phenotypic plasticity. Experimental studies of this skink show that higher-elevation females lay eggs with more developed embryos than is the case for low-elevation conspecifics; but keeping females under cold conditions in captivity also extends the duration of uterine retention of eggs and thus, the stage of embryogenesis at oviposition (Telemeco *et al*., 2010). Climate-associated clines in the degree of embryonic development at the time of laying have been reported in other taxa also, but without experimental evidence on the relative importance of local adaptation and phenotypic plasticity [e.g. Huey, 1977 for Anolis cybotes (Cope 1862)].

In the pet trade, some oviparous squamates may retain their eggs to the point where the eggshell adheres to the oviducal epithelium, if they are not provided with a suitable warm, humid ‘nesting box’ (Mattison, 1998; Mader, 2006). This condition (dystocia) is regarded as a pathology of captive squamates and requires surgical intervention (Wellehan & Gunkel, 2004; Mader, 2006). In summary, some oviparous squamates may be able to retain their eggs for a short amount of time in response to unfavourable climatic conditions for nesting, but their capacity to do so appears limited.

By contrast, transitional forms of viviparity that are reliably present in a species are extremely rare (Shine, 1983; Blackburn, 1995; Shine & Thompson, 2006). Populations of animals displaying consistently transitional forms of egg-laying in the wild are only exhibited by *L. bougainvillii, S. equalis*, and to some extent Z. *vivipara* (Qualls *et al*., 1995; Smith, 1996; Heulin *et al*., 2002). Whether these transitional forms are maintained over a geological timescale is an open question (see Section-V.2), but individuals displaying a transitional reproductive phenotype can be found, year after year, in the same geographic locations. In these three species, the transitional phenotype seems to be genetically determined, rather than the resultof phenotypic plasticity. These lizards are therefore valuable candidates for determining the evolutionary genetics underpinning such evolutionary transitions.

In transitional reproductive forms, mothers produce eggs that are retained internally for long periods, laid at an advanced stage of embryonic development, and then hatch after only a short period of external incubation, compared to other oviparous species. In particular, *S. equalis* and *L. bougainvillii* (see Section IV) have populations that lay thin-shelled eggs at far later stages than other oviparous squamates (Andrews & Mathies, 2000). The rarity of such transitional forms of reproduction may be because they represent a ‘fitness valley’ between oviparity and viviparity, in which the costs of both reproductive modes are incurred (e.g. females are physically burdened for longer than in oviparity, and unable to have a second clutch in the season), but only some of the advantages are gained (e.g. ability to thermoregulate the embryos, but only for part of development), producing an overall unstable life-history strategy (Shine & Bull, 1979; Blackburn, 1995; Smith & Shine, 1997; Shine & Thompson, 2006; Stewart & Blackburn, 2014; Griffith *et al*., 2015; Shine, 2015). Transitional traits such as thinner or less-calcified eggs may be more vulnerable to desiccation, predation, and fungal infection than the thicker, more calcified eggs of truly oviparous individuals (Qualls, 1996; Smith & Shine, 1997; Shine & Thompson, 2006; Griffith *et al*., 2015; Horreo *et al*., 2019). Extended egg retention could also be detrimental to the resulting hatchlings by reducing the time they have available to grow and mature before the season changes (e.g. the onset of winter) (Andrews & Rose, 1994; Shine, 1995). For mothers, while the metabolic cost of pregnancy is negligible for the first third of embryonic development, their oxygen demands increase later in development, which is largely driven by the increasing metabolic rates of the embryos (Van Dyke & Beaupre, 2011), but may also include some metabolic costs of pregnancy (Robert & Thompson, 2000; Foucart *et al*., 2014). In addition, embryonic mass (representing a burden on female locomotion) increases rapidly in the latter part of development, and the increasing embryonic demand for water transport and respiratory gas exchange could result in developmental retardation if these requirements are not met (reviewed in Andrews & Mathies, 2000).

Studying transitional forms along the continuum from oviparity to viviparity can reveal the sequence of morphological and physiological changes that support pregnancy (Qualls, 1996; Smith & Shine, 1997; Andrews & Mathies, 2000; Stewart, 2013). It is therefore important to discover how populations of transitional reproductive forms are generated, despite the apparent disadvantages of such a strategy. In the remainder of this review, we collate information on the biology of transitional reproduction in squamates, before examining alternative hypotheses for the presence of these rare phenotypes in the wild, and suggesting productive future directions for research in this field.

## IV. THE BIOLOGY OF BIMODAL REPRODUCTION AND TRANSITIONAL FORMS

The best-studied bimodally reproductive species are *Z. vivipara*, and to a lesser extent, *L. bougainvillii* and *S. equalis*. All three exhibit transitional forms of viviparity. These species are therefore the focus of this review.

### (1) Eurasian common lizard, Zootoca vivipara

**Fig. 2.**
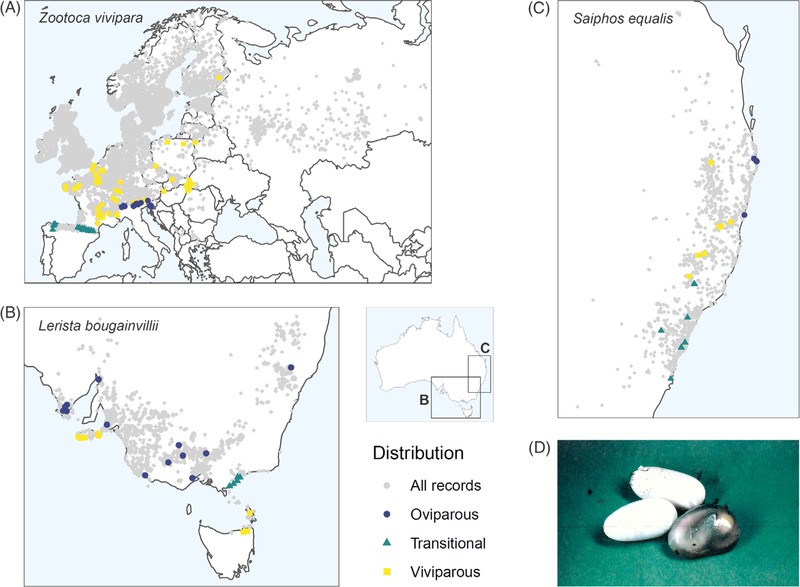
Distribution maps of (A) *Zootoca vivipara* (western Europe) (Global Biodiversity Information Facility, 2021); (B) *Lerista bougainvillii* (Australia) (Atlas of Living Australia, 2021a). (C) *Saiphos equalis* (Australia) (Atlas of Living Australia, 2021b). Distribution of each species is indicated with grey dots, with oviparous, transitional, and viviparous individuals indicated with coloured points for: *Z. vivipara* (Horreo *et al*., 2019), *L. bougainvillii* (Qualls *et al*., 1995) and *S. equalis* (Bustard, 1964; Smith & Shine, 1997; Smith *et al*., 2001). (D) Two eggs from transitional *S. equalis*, alongside a near-term embryo (ventral surface visible) from a viviparous individual (image: S. Smith, used with permission).

*Zootoca vivipara* is the most extensively studied bimodally reproductive vertebrate. It is distributed across Eurasia from Britain to eastern Russia, and most populations are viviparous [clades C, D, E, F (*sensu* Surget-Groba *et al*., 2006; Horreo *et al*., 2018)] (Fig. 2A). Two oviparous populations occur in the southern parts of the range (clade A in the Alps of northern Italy/Slovenia/southern Austria and clade B in the Pyrenees of southern France/northern Spain) (Surget-Groba *et al*., 2006; Horreo *et al*., 2018, 2019). Viviparous embryos are enclosed in thinner shell membranes than are oviparous embryos, probably as a result of the smaller uterine glands of the mothers, and their egg coverings lack the calcareous layer seen in oviparous individuals (Heulin, 1990; Heulin *et al*., 2002, 2005; Stewart, Ecay & Heulin, 2009).

Some populations were recently reclassified as subspecies: *Z. vivipara carniolica* (Mayer *et al*., 2000) (clade A, oviparous), *Z. v. louislantzi* (Arribas, 2009) (clade B, oviparous), and *Z. v. vivipara* (clades C, D, E, F viviparous) (Cornetti *et al*., 2015b). *Zootoca v. vivipara* and *Z. v. carniolica* have small regions of overlap (parapatry), while *Z. v. louislantzi* is geographically isolated (allopatry). The oviparous subspecies display distinct reproductive phenotypes: *Z. v. carniolica* has thicker eggshells and deposits embryos at an earlier embryonic stage (mean 31, range 30–32) which hatch after longer incubation (mean 35 days at 22.5°C) than is the case for *Z. v. louislantzi* (mean 33, range 30–35; 29 days to hatching at 22.5°C) (Heulin *et al*., 2002). *Zootoca v. louislantzi* could represent a transitional form between oviparity and viviparity, although the extended egg retention in this subspecies is not as pronounced as in some populations of *S. equalis* and *L. bougainvillii* (see Sections IV.2 and IV.3).

Several studies failed to find evidence of genetic hybrids between parity modes at contact zones (*Z. v. vivipara* clade A and *Z. v. carniolica* clade E) (Cornetti *et al*., 2015a, b). However, others have found introgression in *Z. vivipara* subspecies between populations of different parity modes (clades A and E at multiple locations), although at much lower levels than between populations with the same parity mode [within clade B at multiple locations; clades D and E at multiple locations (Horreo *et al*., 2019)], and in small numbers [~6% of sampled individuals (Recknagel *et al*., 2021a); two putative ‘hybrids’ (Lindtke, Mayer & Böhme, 2010)]. Therefore, there is some degree of reproductive isolation between oviparous and viviparous populations in the wild. There are also karyotypic differences between some clades. For example, the karyotype of oviparous clade A (*Z. v. carniolica*) is 2*n* = 36 for both sexes, with the ancestral ZW sex-determination system (Recknagel, Kamenos & Elmer, 2018), as is the case for one viviparous lineage (*Z. v. vivipara* clade F), whereas other viviparous lineages (*Z. v. vivipara* clades C, D, and E) and oviparous clade B (*Z. v. louislantzi*) have n*2* = 35 chromosomes for females and 2*n* = 36 for males, and sex determination by Z_1_Z_2_W, with differing centromere locations between lineages (Odierna *et al*., 2004; Kupriyanova, Kuksin & Odierna, 2008; Kupriyanova, Kirschey & Bohme, 2017).

Could *Z. v. vivipara*, *Z. v. carniolica*, and *Z. v. louislantzi* be separate species? Several facts support this view: there are (*i*) few naturally occurring putative phenotypic ‘hybrids’ between *Z. v. vivipara* and *Z. v. carniolica* in their contact zone and no evidence of genetic hybrids; (*ii*) karyotypic differences between lineages, which could prevent correct assortment of chromosomes at meiosis; and (*iii*) differences in reproductive phenotype between *Z. v. vivipara* and *Z. v. carniolica/louislantzi*. On the other hand, there is evidence that the lineages are not separate species, suggesting that the subspecies designation is appropriate: (*i*) *Z. v. vivipara* and *Z. v. carniolica* are genetically distinct to approximately the subspecies level (Mayer *et al*., 2000); (*ii*) oviparous and viviparous forms of *Z. vivipara* are non-monophyletic (Horreo *et al*., 2018), and (*iii*) reproductive isolation between the subspecies is incomplete (e.g. Horreo *et al*., 2019).

The allopatric *Zootoca v. vivipara* and *Z. v. louislantzi* have been crossed in the laboratory (Heulin, Arrayago & Bea, 1989; Arrayago, Bea & Heulin, 1996). No mate choice was offered. These experiments revealed no or only partial reproductive isolation between the two subspecies. The phenotypes of the F_1_ hybrids were intermediate between oviparity and viviparity: thinly shelled and partially calcified eggs, laid at a more advanced stage of development, and incubated for a shorter period before hatching (Heulin *et al*., 1992; Arrayago *et al*., 1996). Unfortunately, to our knowledge, hybridisation between parapatric *Z. v. vivipara* and *Z. v. carniolica* has not been tested in the laboratory. However, the reproductive phenotype of laboratory-generated *Z. v. vivipara* × *Z. v. louislantzi* F_1_ matches the reproductive phenotypes of the two putative *Z. v. vivipara* × *Z. v. carniolica* hybrids (Lindtke *et al*., 2010) and the 6% of genetic hybrids (Recknagel *et al*., 2021b) found in the wild contact zone. Embryo mortality in clutches produced by these wild ‘hybrids’ is high; some of these offspring (which may be F_2_ or backcrosses) appear to be viable, but their fertility is unknown (Lindtke *et al*., 2010). The fact that few hybrid individuals were found in the wild contact zone suggests that hybrids may be disadvantaged compared to the ‘pure’ oviparous and viviparous animals, or that there is sexual selection for mating within a parity mode. Further experiments are required to explore the evolution and distribution of parity mode in *Z. vivipara*, including addressing questions such as: (*i*) can *Z. v. vivipara* and *Z. v. carniolica* hybridise in the laboratory when no mate choice is offered? (*ii*) If they can, then when given mate choice, is there evidence of sexual selection preference for their own subspecies? (*iii*) What is the fitness of hybrid individuals under wild conditions?

### (2) Bougainville's skink, Lerista bougainvillii

*Lerista bougainvillii* is a semi-fossorial skink that lives in south-eastern Australia (Fig. 2B). Morphological analysis, allozyme analysis and mitochondrial DNA (mtDNA) sequence data from a small number of individuals indicates that the various populations of *L. bougainvillii* represent a single species (Qualls *et al*., 1995; Fairbairn *et al*., 1998). *Lerista bougainvillii* is oviparous on the Australian mainland (South Australia, Victoria, and New South Wales) (Greer, 1989; Qualls *et al*., 1995), where it lays shelled eggs at embryonic stages 32–33 (mean 33) (Qualls, 1996), which hatch after ~29 days (at 29°C). On Kangaroo Island, Tasmania, and some Bass Strait islands (Chappell Island, Flinders Island), *L. bougainvillii* is viviparous (Greer, 1989), with offspring born at stage 40 enclosed in thin transparent membranes that are broken at or withina few days of parturition (Qualls *et al*., 1995). There is also a small mainland population of apparently transitional animals in East Gippsland (Victoria) (Qualls *et al*., 1995) that lay partially shelled eggs at stages 35–37 (mean 36), which hatch after ~19 days (at 29°C) (Qualls, 1996). This phenotype shows a much longer duration of egg retention by females than in transitional *Z. v. louislantzi*.

*Lerista bougainvillii* is not as well studied as *Z. vivipara*, but comparisons of clutch size (Qualls & Shine, 1995), some specific costs of reproduction (Qualls & Shine, 1998a), and diet (Barden & Shine, 1994) show few differences between oviparous and viviparous individuals. This species is therefore an ideal model for comparative studies on the evolution of viviparity because the populations seem to differ by very little apart from mode of reproduction. Scanning electron microscopy reveals that the thickness of the shell membrane decreases with increasing duration of egg retention in each population, suggesting that eggshell thinning occurs concomitant with extended retention of eggs (Qualls, 1996). Uterine morphology during gravidity also differs between populations (Adams *et al*., 2007). Viviparity may have arisen twice in *L. bougainvillii* (Qualls *et al*., 1995; but see Fairbairn *et al*., 1998). These origins of viviparity are likely to have been very recent, because Kangaroo Island and the mainland are geographically close and were only separated by sea level rise ~10,000 years ago (Rawlinson, 1974).

### (3) Three-toed skink, Saiphos equalis

*Saiphos equalis* is a nocturnal fossorial skink (Wu, Parker & Thompson, 2009) with a wide range across coastal eastern Australia (Fig. 2C) (Cogger, 2014). It is the only species in its genus and is nested phylogenetically in a clade containing oviparous species (Reeder, 2003; Singhal *et al*., 2018). Phylogenetic inference based on mtDNA strongly indicates a single species (Smith *et al*., 2001), although the work examined few individuals and thus did not have enough resolution to determine population connectivity nor interbreeding between reproductive modes. Similarly, morphological and electrophoretic data support a single species (Smith, 1996). While the reproductive phenotype of the species has not been documented across much of its range, *S. equalis* appears to display at least three reproductive modes (Smith & Shine, 1997; Smith *et al*., 2001): viviparous, oviparous, and transitional.

Viviparous *S. equalis* are born at embryonic stage 40, enclosed within thin transparent membranes that are broken at or shortly after birth (mean 1.5 days, range < 12 h to 7 days) (Smith & Shine, 1997; Smith *et al*., 2001). These individuals tend to live in areas of relatively high elevation (Smith & Shine, 1997). Other *S. equalis* populations, such as those around Sydney, display a transitional phenotype where thinly shelled eggs are laid at advanced embryonic stages (stage 38–39) and briefly incubated externally (mean 5.5 days, range 1 to 7 days) (Smith & Shine, 1997; Smith *et al*., 2001). The difference between stage 38 and stage 40 represents a large proportion of embryonic development, because the later stages progress slowly (Andrews & Mathies, 2000). This transitional form appears to be much better established than the putative ‘wild hybrid’ phenotype of *Z. v. carniolica* and *Z. v. vivipara*, because it is the only reproductive mode that has been observed in these locations over many years of study (e.g. Smith, 1996; Smith & Shine, 1997; Parker *et al*., 2010; Stewart *et al*., 2010; Whittington *et al*., 2015). The eggshells of transitional individuals have a higher concentration of calcium than egg coverings of viviparous embryos (Linville *et al*., 2010) and are also more opaque and thicker (Fig. 2D), probably due to a higher density of maternal uterine shell glands in oviparous individuals (Stewart *et al*., 2010). Finally, a third phenotype is displayed by at least one coastal population that oviposits shelled eggs that are incubated externally for a much longer period (15 days) than transitional embryos (Smith *et al*., 2001). Although eggshell thickness has never been quantified and the embryonic stage at parition is currently unknown for this population, this phenotype could be close to ‘normal’ oviparity.

*Saiphos equalis* are morphologically similar across the range (Smith, 1996), and clutch size and mass are similar between the transitional and viviparous individuals tested so far (Smith & Shine, 1997). Experimental exposure of transitional and viviparous animals to different environmental conditions does not change reproductive mode, suggesting that variation in reproductive mode is heritable and not a result of phenotypic plasticity, at least in the short term (Smith, 1996). Intriguingly, *S. equalis* is the only vertebrate in which a mixed parity mode has been observed *within an individual* – albeit only a single animal (Laird, Thompson & Whittington, 2019). The incubation periods for individuals in this species vary wildly even within parity mode. Thus *S. equalis* reproductive variation needs to be quantified at a much finer scale to determine the number of origins of viviparity in this species.

## V. WHAT PROGESSES PRODUCE REPRODUCTIVE BIMODALITY AND TRANSITIONAL FORMS WITHIN A SPEGIES?

### (1) Reproductive bimodality

*Lerista bougainvillii*, *S. equalis*, and *Z. vivipara*, remarkably, display at least three different heritable reproductive phenotypes each. How is such phenotypic variability produced? Variation in reproductive phenotype within each species could result from, and then reinforce, local adaptation across the range. For example, an ecological shift in one area of the species’ range might drive the allopatric evolution of viviparity in that population. Once differences in parity mode are established, if secondary contact as a result of an ecological shift takes place, the genetic differences between populations might already be so great that reproductive isolation results (Horreo *et al*., 2019). This scenario is plausible in explaining the distribution of *Z. v. carniolica* (oviparous) and *Z. v. vivipara* (viviparous): perhaps some originally oviparous *Z. vivipara* persisted in warm refugia during the Pleistocene, whilst others dispersed to disjunct populations in cooler climates evolving viviparity in allopatry; after further ecological change, secondary contact occurred, with *Z. v. carniolica* and *Z. v. vivipara* now experiencing a degree of reproductive isolation resulting in the co-occurrence of oviparity and viviparity in the contact zone (Surget-Groba *et al*., 2001; Cornetti et al., 2015b). Similar processes may explain the phenotypic variation in other bimodally reproductive species, although this possibility remains to be investigated.

The degree of reproductive isolation between the reproductive phenotypes of bimodal species, and the mechanisms underlying it if present, are mostly unstudied, except for *Z. vivipara*. There are many plausible mechanisms that should be tested. (*i*) Chromosomal rearrangements could explain reproductive isolation: for example, the differences in chromosomal complement and sex determination within *Z. vivipara* could easily result in failure at meiosis for some directions of crosses between individuals with different karyotypes (Horreo *et al*., 2019). (*ii*) Crosses between parents of different parity modes, even if not completely incompatible, could result in outbreeding depression, e.g. if local adaptation results in a mismatch between embryonic requirements and maternal provisioning (Lindtke *et al*., 2010; Horreo *et al*., 2019), or incompatibility between a hybrid embryo and viviparous mother due to close tissue associations and the potential for genomic conflict (Zeh & Zeh, 2000). (*iii*) Pre- or post-copulatory sexual selection might result in assortative mating within a parity mode, as a result of small differences in morphology and mate preferences (Horreo *et al*., 2019). Such mechanisms could also explain the low prevalence of wild hybrids in *Z. vivipara* contact zones, but have not been explored in other bimodal species.

### (2) Transitional forms of pregnancy

There are several processes that could produce an intermediate reproductive form between oviparity and viviparity. These mechanisms include: gradual evolutionary change via selection on oviparous individuals for increasing duration of egg retention (see Section III.2); hybridisation between egg-laying and live-bearing individuals (Fairbairn *et al*., 1998); and ‘reversals’ from viviparity to oviparity.

#### (a) Hybridisation

Hybridisation is a plausible source of transitional forms, because laboratory crossbreeding between oviparous and viviparous *Z. vivipara* results in intermediate reproductive traits: a long embryo retention time and irregular less-calcified eggshell structure (Arrayago *et al*., 1996). In *Z. vivipara*, contemporary introgression between parity modes to produce the persistently observed intermediate form (*Z. v. louislantzi*) can be ruled out: *Z. v. louislantzi* is disjunct from other reproductive modes (Fig. 2), and diverged from the viviparous (*Z. v. vivipara*) form >2 Mya (Horreo *et al*., 2018). While contemporary hybridisation could produce transitional forms of *S. equalis* and *L. bougainvillii*, this scenario is only plausible if there are undiscovered oviparous and viviparous forms close to transitional locations. Laboratory hybridisation studies and fine-scale mapping of reproductive phenotype represent important areas for future research in these species. To summarise, if hybridisation has produced persistent populations of transitional forms of pregnancy in the wild, it is most likely to have been a historical event (or events).

#### (b) Reversals from viviparity to oviparity

Transitional forms could also result from ‘reversals’ from viviparity back to oviparity. Whether oviparity can re-evolve from viviparity in squamates has been the subject of much debate (e.g. Pyron & Burbrink, 2014; Griffith *et al*., 2015; Shine, 2015; Blackburn, 2015b; Horreo, Suarez & Fitze, 2020), and is beyond the scope of this review. To summarise the arguments, reversals may be uncommon because of lowered fitness (Shine, 2015) or because of the complex anatomical and physiological requirements in regaining the ability to produce eggs, including uterine shell glands, coordinated uterine musculature to rotate the egg during shell deposition, nesting behaviour, and altered timing of reproductive processes including calcium transport to form the eggshell (e.g. Griffith *et al*., 2015). While there is debate around putative squamate parity mode reversals identified via ancestral state reconstruction, there is some evidence for ‘re-evolution’ of oviparity in several oviparous taxa that are deeply nested in viviparous clades (e.g. Lynch & Wagner, 2010; Esquerré *et al*., 2019), although an alternative less-parsimonious explanation is that there are multiple independent origins of viviparity among taxa in which ancestral oviparity is retained.

We posit that reversals are plausible in bimodally reproductive species because viviparity is of recent origin. While the genetic machinery underlying the production of shelled eggs is likely to have been lost over time in species with ancient origins of viviparity (Griffith *et al*., 2015), the biological requirements for oviparity are less likely to have been completely lost in recent origins, and may perhaps require only a few genetic changes to be regained. It is possible that transitional reproductive forms are a result of transitions ‘back’ from viviparity. One reversal from viviparity to oviparity is already strongly supported in *Z. vivipara* (Surget-Groba *et al*., 2006; Recknagel *et al*., 2018; Horreo *et al*., 2020). The most parsimonious explanation of the incomplete phylogeny of *S. equalis* also indicates a reversal leading to the transitional form (Smith *et al*., 2001). The co-occurrence of oviparity and viviparity within a single *S. equalis* individual from an ordinarily viviparous population could also indicate that the requirements to produce an egg are maintained in recent origins of viviparity (Laird *et al*., 2019). Well-supported phylogenetic evidence would definitively indicate reversals; fortunately, it is easier to reconstruct more robust phylogenies within than among species, so squamates with intraspecific variation in reproductive mode are ideal places to look for reversions from viviparity to oviparity. If we accept that reversals are rare, if they have produced transitional forms, these events are also likely to have been historical (rather than repeated reversals to produce transitional forms year after year).

#### (c) Are transitional forms transient or persistent?

The existence of transitional forms of pregnancy is puzzling given that this phenotype theoretically represents a ‘fitness valley’ between oviparity and viviparity. Are such transitional forms transient, and we are serendipitously present to observe them today, or might these transitional forms be maintained? Transitional forms have been repeatedly observed in the wild in the same geographic locations, and thus appear to be maintained over timeframes of at least several decades. Over geological timescales though, transitional forms should be transient if they truly represent a fitness valley. Alternatively, transitional phenotypes might persist for long periods if they are more advantageous than oviparity or viviparity under certain environmental conditions, for example as a bet-hedging strategy in variable environments [both live birth and egg-laying have been observed in a single pregnancy in one *S. equalis* in the laboratory, so parity mode could be labile (Laird *et al*., 2019)]. Such open questions make transitional forms even more interesting for evolutionary studies.

## VI. GENETIC AND GENOMIC TOOLS TO INVESTIGATE REPRODUCTIVE VARIATION WITHIN A SPECIES

Genetic and genomic tools can provide fine-scale knowledge of population structure and interconnectivity to map transitions within a species from oviparity to viviparity (and potentially back to oviparity), estimate divergence dates for these transitions, and determine how reproductive variation is produced within a species. There are two broad approaches. First, population genetics can be used to reconstruct histories of differentiation among populations to determine evolutionary mechanisms shaping diversity at the landscape scale. Second, molecular genetics can be used to determine the genetic basis of phenotypic variation among individuals and populations, providing hypotheses around genomic mechanisms of population differentiation.

From a population genetic perspective, sampling of animals from across the species range using a genome-wide survey method such as reduced representation sequencing (e.g. Peterson *et al*., 2012) enables the reconstruction of historic and contemporary population dynamics, which can inform the mechanisms of evolution of parity modes. For bimodal species, important studies of this type have been conducted in 40 *Z. vivipara* representing five major European clades and three subspecies with parity mode variation (Cornetti *et al*., 2018), and ~ 800 *Z. vivipara* at the *Z. v. vivipara* and *Z. v. carniolica* contact zone (Recknagel *et al*., 2021a). These studies detected no and limited hybridisation (~6%) across subspecies (parity modes), respectively. It remains to be seen whether similar discrimination is observed in other bimodal species.

Molecular genetic techniques, supported by a high-quality genome, offer the opportunity to determine the mechanistic basis of different parity modes within a species. Parity mode may be controlled by differences in genome sequence, structure, and gene regulation (Ecker *et al*., 2018). The genome sequence of viviparous *Z. v. vivipara* (Yurchenko, Recknagel & Elmer, 2020) is an important resource for genomic comparisons within a bimodally reproductive species. Gene regulation can be measured indirectly by examining gene expression, and transcriptomic studies of uterine gene expression of transitional and viviparous *S. equalis* (Foster *et al*., 2020) and oviparous and viviparous *Z. vivipara* (Recknagel *et al*., 2021a) have already found differences in the expression of thousands of genes between parity modes.

Combining population genetic techniques with genomic information will be a powerful method for exploring the mechanistic basis of parity mode evolution in the future. For example, examining genomic evidence for introgression can determine which parts of the genome experience the greatest selection and may produce new phenotypes in new environments. Recknagel *et al*.’s (2021a) study of *Z. vivipara*, which identified genomic regions associated with eggshell traits and gestation time and regions under selection between viviparous and oviparous individuals, is an excellent example of the utility of combining population genetic and genomic methods.

## VII. CONCLUSIONS

1. The evolution of pregnancy from egg-laying has involved changes to behaviour, physiology, and morphology, underpinned by genetic change, and is an ideal model for understanding the evolution of phenotypic innovation. Viviparity has evolved independently more than 150 times in vertebrates alone, and some extant species exhibit transitional forms, which is uncommon for such traits generally.
2. Although intraspecific variation in reproductive mode is rare, four bimodally reproductive species of vertebrates offer the opportunity for comparative research into the genetics, physiology, and evolution of pregnancy by removing many of the confounding factors implicit in interspecific comparisons.
3. In three bimodally reproductive lizards, the co-occurrence of oviparity, viviparity, and a transitional form of extended retention of eggs inside the uterus (particularly in *L. bougainvillii* and *S. equalis*) offers a unique opportunity for comparative evolutionary research to understand the transition between reproductive modes and the evolution of pregnancy.
4. Application of molecular and genetic tools to these species promises to clarify mechanisms underpinning the maintenance of multiple reproductive modes within a species, including transitional forms. A combination of genomic and population genetic approaches is recommended.
